# Fluorimetric methods for the measurement of intermediate metabolites (lactate, pyruvate, alanine, β-hydroxybutyrate, glycerol) using a COBAS FARA centrifugal analyser

**DOI:** 10.1155/S1463924693000240

**Published:** 1993

**Authors:** L. D. Monti, P. E. Sandoli, S. Costa, V. C. Phan, P. M. Piatti

**Affiliations:** Laboratorio Metabolismo Intermedio L15 Istituto Scientifico San Raffaele University of Milano Via Olgettina 60 Milano 20132 Italy

## Abstract

Intermediate products of the metabolism of glucose, fat and amino-acid are important in the evaluation of such metabolic disorders as diabetes mellitus, liver disease and metabolic acidosis. In the present study, methods for the measurement of intermediate metabolites (lactate, pyruvate, alanine, β-hydroxybutyrate and glycerol) have been adapted to a fast centrifugal analyzer: the COBAS FARA. Correlation coeffcients rangedfrom 0.90 to 0.99, compared to established manual spectrophotometric methods. Within-run coeffcients of variation (CVs) ranged between 2.9 and 8.8% at low levels, between 1.5 and 5.7% at medium levels and between 1.2 and 5.6% at high levels. Between-run CVs were between 4.0 and 15.0% at low levels, between 1.7 and 7.0% at medium levels and between 1.3 and 2.7% at high levels. These fluorimetric assays for the determination of intermediate metabolites on COBAS FARA (Roche) have a good sensitivity and precision, are less costly than manual methods and can be used on a routine basis.


Journal of Automatic Chemistry, Vol. 15, No. 5 (September-October 1993), pp. 177-181

### Fluorimetric methods for the measurement of intermediate metabolites (lactate, pyruvate, alanine, β-hydroxybutyrate, glycerol) using a COBAS FARA centrifugal analyser

**L. D. Monti, P. E. Sandoli, S. Costa, V. C. Phan and P. M. Piatti**

*Laboratorio Metabolismo Intermedio L15, Istituto Scientifico San Raffaele, University of Milano, Via Olgettina 60, 20132, Milano, Italy*

*Intermediate products of the metabolism of glucose, fat and amino-acid are important in the evaluation of such metabolic disorders as diabetes mellitus, liver disease and metabolic acidosis. In the present study, methods for the measurement of intermediate metabolites (lactate, pyruvate, alanine, β-hydroxybutyrate and glycerol) have been adapted to a fast centrifugal analyzer: the COBAS FARA. Correlation coefficients ranged from 0·90 to 0·99, compared to established manual spectrophotometric methods. Within-run coefficients of variation (CVs) ranged between 2·9 and 8·8% at low levels, between 1·5 and 5·7% at medium levels and between 1·2 and 5·6% at high levels. Between-run CVs were between 4·0 and 15·0% at low levels, between 1·7 and 7·0% at medium levels and between 1·3 and 2·7% at high levels. These fluorimetric assays for the determination of intermediate metabolites on COBAS FARA (Roche) have a good sensitivity and precision, are less costly than manual methods and can be used on a routine basis.*

### Introduction

The measurement of intermediate metabolism products was used in the past to understand such metabolic disorders as diabetes mellitus, liver disease and metabolic acidosis [1-5]. They are now measured to evaluate early metabolic alterations in certain classes of patients, for example obese patients and relatives of diabetic subjects who are at risk for developing metabolic disorders. Enzymatic assays for intermediate metabolites were devised in the 1950s [6], and manual methods for the determination of intermediate metabolites (lactate, pyruvate, alanine, β-hydroxybutyrate and glycerol) were rapidly developed for the study of physiological and pathological states [7-11], however, these methods are laborious and have little sensitivity. They are also expensive, as they use relatively large quantities of enzymes with little substrate to enable the reaction to proceed rapidly to equilibrium. In order to analyse large numbers of samples rapidly in small volumes of blood or plasma, automated photometric [12] or fluorimetric techniques [13 and 14] have been developed utilizing either continuous flow methods [15] or centrifugal analysers with a fluorimetric attachment [16-18]. These methods are more precise and sensitive and use very little sample, thus making metabolic studies possible in children and neonates. The present study started from the need to use fluorimetric methods with assays particularly suited to routine work. The measurement of lactate, β-hydroxybutyrate, alanine, pyruvate and glycerol was developed on a fast centrifugal analyser (COBAS FARA).

### Materials and methods

### Patients

Fifty subjects were subdivided into three groups: normal; impaired glucose tolerant; and diabetic patients attending a diabetic clinic. Each patient had a random blood sample for the determination of intermediate metabolites. They were chosen because they had a wide concentration range of all metabolites. Blood samples were processed simultaneously using the COBAS FARA methods and manual methods described in the literature [7-11].

### Sample preparation

Lactate, β-hydroxybutyrate, alanine, pyruvate and glycerol are unstable in whole blood and an immediate and correct processing of the sample is necessary at the time of venepuncture [15]. Plastic tubes containing 3 ml of 5% (0·5 mol/l) perchloric acid (PCA), previously cooled to 0°C, were weighed; 1-1.5 ml of blood was added immediately after sampling and the tube was weighed again, thus giving an accurate measurement of the amount of blood used. In a few pathological cases, whether the levels of metabolites were over the highest calibrator points, dilutions were made adding adequate amounts of 5% PCA. All these changes were taken into account in the final calculation of the data.

### Comparison of methods

The methods of measurement for lactate, pyruvate, alanine, β-hydroxybutyrate and glycerol were adapted for use on the COBAS FARA II (Roche, Basle, Switzerland) with a fluorimetric attachment by making a number of modifications in the composition of buffers, and coenzyme/enzyme reagents used in previous methods [16] and modifying the reaction modes available on the analyser to take the improved calculation programs into account.

In order to compare the methods under study, manual spectrophotometric assays [7-11] were performed with a

0142-0453/93 $10.00 © 1993 Taylor & Francis Ltd.

550 SE UV/VIS spectrophotometer (Perkin-Elmer). In this case, samples were precipitated with PCA but the dilution ratio was 1:1.

### Reagents

NAD (free acid, grad I 100%, cat. 127965), NADH (disodium salt, grad I 100%, cat. 837075), lactat-dehydrogenase 100 mg (10 ml) cat. 107085, β-hydroxy-butyrate-dehydrogenase 25 mg (5 ml) cat. 127841, L-alanin-dehydrogenase (150 U) cat. 102636, glycero-kinase 5 mg (1 ml) cat. 127795, glycerin-3-phosphat-dehydrogenase 10 mg (1 ml) cat. 127752, ATP cat. 519979, β-hydroxybutyrate (monosodium salt), pyruvate (monosodium salt), and L-alanine (crystallized) were all supplied by Boehringer Mannheim GmbH, Germany. Lactic acid (lithium salt) and glycerol were supplied by the Sigma Chemical Co., PO Box 14508, St. Louis, MO 63178, USA.

### Assay procedures

*Lactate.* Buffer: 0·5 mol/l glycine, 1·6 mol/l hydrazine hydrochloride, adjusted to pH 9·6 with 10 mol/l sodium hydroxide (reagent solution). Enzyme reagent: 50 mg NAD and 500 µl lactate dehydrogenase in 3·5 ml 0·1 mol/l phosphate buffer, pH 7·4 (starter solution). Calibrators: 0, 625, 125, 250, 500, 750, 1000 µmol/l. Dilutions of the calibrator curve were made using PCA 3%.

The PM voltage was set using 1200 µmol/l calibrator.

The program was set by introducing the following variables: calibration mode: logit/log 4; sample volume: 5·0 µl; reagent solution: 270 µl; starter solution: 20 µl; incubation time: 310 s; MI: 300 s; number of readings: 10; interval between readings: 30 s; calculation step: endpoint (first MI, last 10).

*β-hydroxybutyrate.* Buffer: 0·1 mol/l TRIS, 1 mol/l hydrazine hydrate, 2·7 mol/l EDTA (disodium salt) adjusted to pH 8·5 with 10 mol/l hydrochloric acid. Enzyme reagent: 10 mg NAD and 350 µl β-hydroxybutyrate dehydrogenase in 5·0 ml 0·1 mol/l phosphate buffer, pH 7·4. The reagent solution is prepared by adding 0·7 ml of this solution to 8·8 ml of the buffer. Calibrators: 12.5, 25, 50, 75, 100, 200, 300 µmol/l. Dilutions of the calibrator curve were made using PCA 3%.

The PM voltage was set using top calibrator (300 µmol/l).

The program was set by introducing the following variables: calibration mode: linear regression; sample volume: 20 µl; reagent volume: 270 µl; number of readings 20; interval between readings: 30 s; calculation step: endpoint (first 1, last 20).

*Alanine.* Buffer: 0·04 mol/l TRIS, 1 mol/l hydrazine hydrate, 1·34 mmol/l EDTA (disodium salt) adjusted to pH 10·0 with 10 mol/l hydrochloric acid.

Enzyme reagent: 20 mg NAD and 100 µl alanine dehydrogenase in 10 ml 0·1 mol/l phosphate buffer pH 7·4. The reagent solution is prepared by adding 0·7 ml of this solution to 8·8 ml of the buffer.

Calibrators: 50, 75, 100, 150, 300 µmol/l. Dilutions of the calibrator curve were made using PCA 3%.

The PM voltage was set using top calibrator (300 µmol/l).

The program was set by introducing the following variables: calibration mode: linear regression; sample volume: 10 µl; reagent volume: 270 µl; number of readings: 20; interval between readings: 30 s; calculation step: endpoint (first 1, last 20).

*Pyruvate.* Buffer: 0·4 mol/l triethanolammonium chloride, 10 mmol/l disodium EDTA, adjusted to pH 7·4 with 10 mol/l sodium hydroxide. Coenzyme reagent: 1 mg NADH in 1 ml of 0·1 mol/l phosphate buffer pH 7·4, the final reagent is prepared by adding 100 µl of this solution to 8 ml of the buffer (reagent solution).

Enzyme reagent: 25 µl lactate dehydrogenase is added to 2 ml 0·1 mol/l phosphate buffer, pH 7·4 (starter solution).

Calibrators 12·5, 25, 50, 75, 100 µmol/l. Dilutions of the calibrator curve were made using PCA 3%.

The PM voltage was set using water as sample.

The program was set by introducing the following variables: calibration mode: linear regression; sample volume: 10 µl; reagent solution: 170 µl; starter solution: 10 µl; incubation time: 300 s; number of readings: 10; interval between readings: 30 s; calculation step: endpoint (first 1, last 10).

*Glycerol.* Buffer: 0·2 mol/l glycine, 1 mol/l hydrazine hydrate, 10 mmol/l magnesium chloride brought to pH 9·5 with 10 mol/l sodium hydroxide.

Enzyme reagent: 3 mg NAD, 3 mg ATP, 15 µl glycerokinase and 30 µl glycerol-3-phosphate dehydrogenase in 1 ml of the pyruvate buffer pH 7·4.

The final reagent solution is prepared by adding 1 ml of this reagent to 8·8 ml of the buffer.

Calibrators: 12·5, 25, 50, 75, 100 µmol/l. Dilutions of the calibrator curve were made using PCA 3%.

The PM voltage was set using 150 µmol/l calibrator.

The program was set by introducing the following variables: calibration mode: linear regression; sample volume: 10 µl; reagent solution: 270 µl; number of readings: 12; interval between readings: 30 s; calculation step: endpoint (first 1, last 12).

### Statisticl analysis

Data are presented as mean ±SD.
Data were processed by least-squares regression analysis.

### Results

Typical calibration curves obtained for the five intermediate metabolites with the COBAS FARA II are reported in figure 1. Good repeatability of the calibration curves was found for all the metabolites, collecting data from 15 calibration curves over a period of three months. CVs were always higher at the lowest points of the

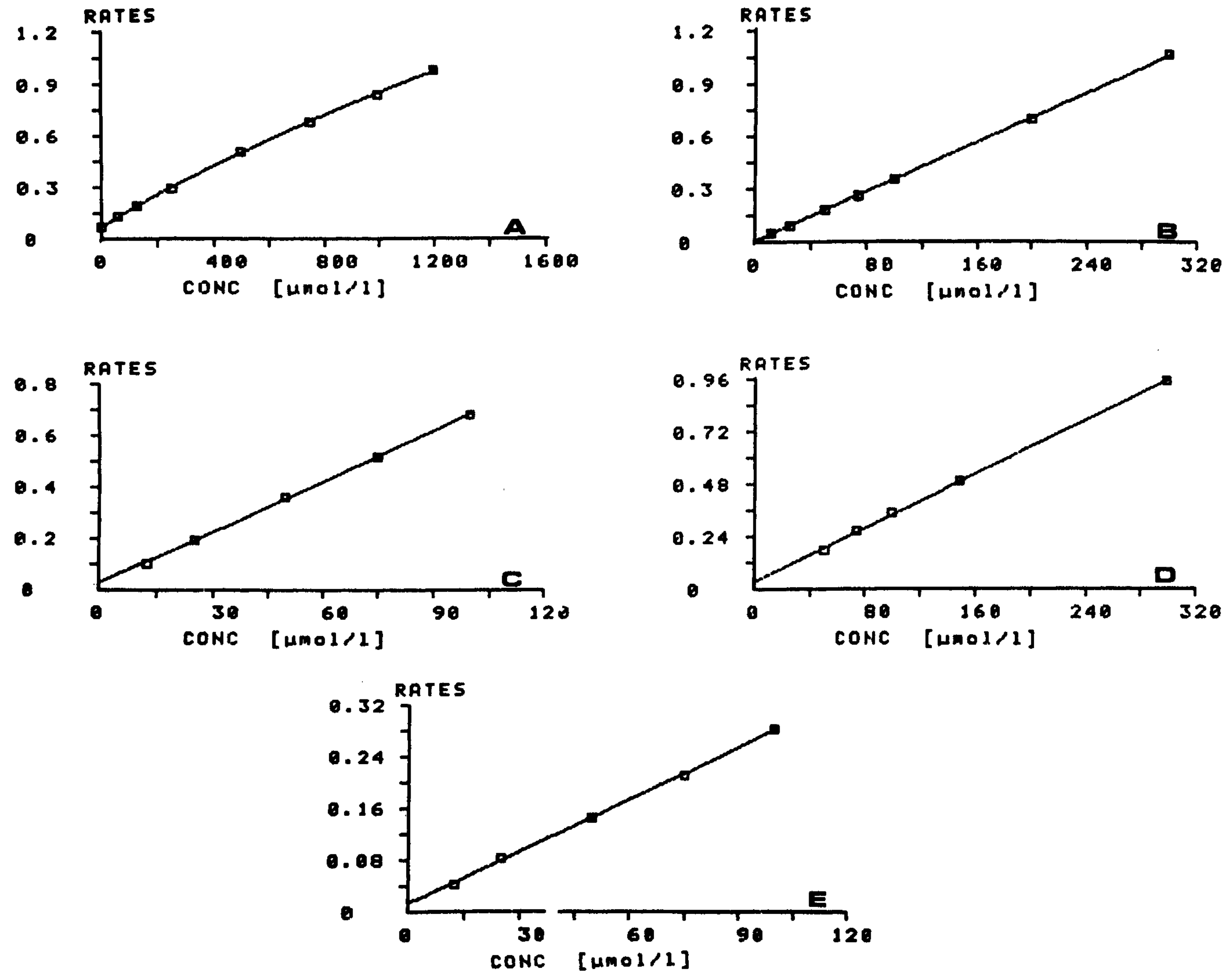

*Figure 1. Calibration curves of lactate (A), β-hydroxybutyrate (B), glycerol (C), alanine (D) and pyruvate (E).*

*Table 1. Coefficients of variation (CVs) of the calibration curves.*

| | Lowest points (range) | Highest points (range) |
|---|---|---|
| Lactate | 3·6–5·4% | 0·6–2·4% |
| β-oh-but | 4·3–9·6% | 0·6–1·4% |
| Glycerol | 1·1–3·8% | 1·1–3·8% |
| Alanine | 0·5–2·9% | 0·5–2·9% |
| Pyruvate | 4·2–7·6% | 1·3–2·8% |

calibration curves; better values were found at the highest points of the calibration curves (table 1).

Analytical recovery was made after adding a calibrator (low, medium or high) to three unknown samples. Average analytical recovery (%) (±SD) was 100·1 (0·6) for lactate, 98·5 (2·6) for β-hydroxybutyrate, 95·8 (2·7) for alanine, 101·0 (5·3) for pyruvate and 100·3 (1·0) for glycerol.

The precision of the fluorimetric methods on the COBAS FARA II was tested by performing within-run and between-run CVs of PCA samples: the results are reported in table 2. Within- and between-run CVs were within an acceptable range in all cases except pyruvate, suggesting a high precision of the methods.

The methods correlated well with manual methods (see table 3); a very small positive intercept, not significantly different from zero, was obtained in all cases except for β-hydroxybutyrate. This is probably because manual spectrophotometric methods are less sensitive than fluorimetric methods at low concentrations of analytes.

### Discussion

In this study fluorimetric methods for the determination of intermediate metabolites (lactate, pyruvate, alanine, glycerol, β-hydroxybutyrate) have been adapted to the COBAS FARA, a fast centrifugal analyser. In the past, intermediate metabolites were assayed using either manual spectrophotometric methods or fluorimetric methods [7–11, 16–19]. When compared to manual methods, the methods described are quicker (5–10 min. versus 30 min.) and cheaper (with savings of more than 100% in terms of enzyme and coenzyme costs). They are

*Table 2. Within-run and between-run CVs (%) from control samples (μmol/l).*

| | | Within run ($N = 30$) | | | | Between run | | |
|---|---|---|---|---|---|---|---|---|
| | | Low | Medium | High | | Low | Medium | High |
| Lactate | Expected value | 62·5 | 125·0 | 500·0 | $N$ | 44 | 26 | 20 |
| | Mean | 61·4 | 128·7 | 500·3 | Mean | 62·7 | 128·9 | 501·5 |
| | CV | 3·6 | 3·2 | 2·3 | CV | 6·1 | 4·6 | 4·3 |
| β-oh-but | Expected value | 25·0 | 75·0 | 100·0 | $N$ | 23 | 44 | 18 |
| | Mean | 23·9 | 74·0 | 98·7 | Mean | 24·4 | 74·5 | 98·4 |
| | CV | 3·8 | 2·1 | 2·0 | CV | 4·0 | 1·7 | 2·7 |
| Glycerol | Expected value | 12·5 | 25·0 | 50·0 | $N$ | 61 | 36 | 20 |
| | Mean | 11·4 | 24·8 | 51·0 | Mean | 11·3 | 25·1 | 51·3 |
| | CV | 3·8 | 1·5 | 1·2 | CV | 5·3 | 2·7 | 1·3 |
| Alanine | Expected value | 50·0 | 75·0 | 100·0 | $N$ | 33 | 35 | 20 |
| | Mean | 44·9 | 73·6 | 95·9 | Mean | 45·8 | 74·0 | 97·2 |
| | CV | 2·9 | 1·9 | 2·0 | CV | 4·8 | 3·3 | 2·2 |
| Pyruvate | Expected value | 12·5 | 25·0 | 75·0 | $N$ | 62 | 30 | 20 |
| | Mean | 13·6 | 23·8 | 72·7 | Mean | 13·5 | 24·3 | 73·4 |
| | CV | 8·8 | 5·7 | 5·6 | CV | 15·0 | 7·0 | 6·3 |

*Table 3. Correlation data of methods on COBAS FARA and manual methods in 50 samples (μmol/l).*

| | $R$ | $S_{xy}$ | Slope | C.I. | Interpret | Mean lower case | Mean upper case | Range (min) | Range (max) |
|---|---|---|---|---|---|---|---|---|---|
| Lactate | 0·99 | 75·2 | 0·98 | 0·93–1·03 | 17·9 | 747 | 752 | 150 | 2390 |
| β-oh-but | 0·99 | 19·8 | 0·98 | 0·94–1·02 | 14·2 | 127 | 138 | 26 | 1000 |
| Alanine | 0·98 | 32·3 | 1·02 | 0·94–1·10 | 9·4 | 341 | 359 | 61 | 577 |
| Pyruvate | 0·99 | 4·2 | 0·96 | 0·90–1·02 | 2·9 | 96 | 95 | 50 | 171 |
| Glycerol | 0·90 | 17·5 | 1·03 | 0·82–1·24 | 0·44 | 66 | 69 | 23 | 200 |

X = COBAS FARA.
Y = Spectrophotometric methods.
C.I. = Confidence interval.

also more precise (the between-run CV was 4·0% at 24·4 μmol/l compared with 10% at 44·0 μmol/l using the method of Hansen and Freier for β-hydroxybutyrate). The high sensitivity of the methods is illustrated by the fact that the reaction sample volumes usually varied from 5 to 10 μl of PCA precipitates with an even smaller volume of blood.

Continuous flow methods [15] were also used for the determination of the intermediate metabolites, but these methods presented some disadvantages when compared to fast centrifugal analyser: for example, the need to run 'blank fluorescence' and to draw calibration graphs and compare large numbers of peaks which can generate gross errors [16]. The present methods on COBAS FARA II are similar to a few spectrophotometric or fluorimetric methods adapted for previous fast centrifugal analysers [16, 19]. Major innovations are in the ability to create new reaction modes with mathematical modeling of the data and particular care in the handling of samples. In conclusion, the fluorimetric assays for the determination of intermediate metabolites on COBAS FARA II have a good sensitivity and precision, are less costly than manual methods and can be used on a routine basis.

### References

1. Alberti, K. G. M. M. and Hockaday, T. D. R., *British Medical Journal*, **2** (1972), 565.

2. Marbach, E. P., *Clinical Chemistry*, **21** (1975), 113.

3. Foster, K., Alberti, K. G. M. M. and Hinks, L., *Clinical Chemistry*, **26** (1978), 1710.

4. Noy, G. A. and Alberti, K. G. M. M., *Rec. Adv. Clin. Biochem.*, **2** (1978), 229.

5. Noy, G. A., Buckle, A. L. J. and Alberti, K. G. M. M., *Clinica Chimica Acta*, **89** (1978), 135.

6. Greengard, P., *Nature*, **178** (1956), 632.

7. Gutman, I and Wahlefeld, A. W., in Bergmeyer, H. U. (Ed.), *Methods of Enzymatic Analysis 4*, 2nd edn, (Academic Press, New York, 1974), 1464.

8. Williamson, D. H. and Mellanby, J., in Bergmeyer, H. U. (Ed.), *Methods of Enzymatic Analysis 3*, 2nd edn, (Academic Press, New York, 1974), 1836.

9. Wieland, O., in *Methods of Enzymatic Analysis 3*, op. cit., 1404.

10. Williamson, D. H., in *Methods of Enzymatic Analysis*, *Vol. 3*, op. cit., 1679.

11. Czok, R. and Lamprecht, W., in *Methods of Enzymatic Analysis 3*, op. cit., 1446.

12. Asrow, G., *Analytical Biochemistry 1*, **28** (1969), 130.

13. Antonis, A., Clark, M., Pilkington, T. R. E., *Journal of Laboratory Clinical Medicine*, **68** (1966), 340.

14. Ozand, P. T., Hawkins, R. L., Collins, R. M., Jr., Tilton, J. T. and Cornblath, M., *Biochemical Medicine*, **14** (1975), 170.

15. Lloyd, B., Burrin, J. M., Smyth, P. and Alberti, K. G. M. M., *Clinical Chemistry*, **24** (1978), 1724.
16. Harrison, J., Hodson, A. W., Skillen, A. W., Stappenbeck, R., Agius, L. and Alberti, K. G. M. M., *Journal of Clinical Chemical Biochemical*, **26** (1988), 141.
17. Moore, J. J., Marcus, M. and Sax, S. M., *Clinical Chemistry*, **28**, (1982), 7023.
18. Pesce, M. A., Bodourian, S. M. and Nicholson, J. F., *Clinical Chemistry*, **27** (1975), 633.
19. Hansen, J. L. and Freier, E. F., *Clinical Chemistry*, **24** (1978), 475.

